# How data drift impacts the safety and interpretability of machine learning models predicting risk from blood glucose control

**DOI:** 10.1136/bmjdhai-2025-000269

**Published:** 2026-02-09

**Authors:** Ho-Hin Leung, Christopher Duckworth, Dan Burns, Matthew Guy, Michael Boniface

**Affiliations:** 1 School of Physics and Astronomy, University of St Andrews, St Andrews, UK; 2 IT Innovation Centre, University of Southampton, Southampton, UK; 3 School of Computer Science, University of Bristol, Bristol, UK; 4 Paediatric Diabetes, University Hospital Southampton NHS Foundation Trust, Southampton, UK

**Keywords:** Data Science, Machine Learning, Preventive Medicine, Artificial intelligence, Disease Management

## Abstract

**Objective:**

Predictive algorithms trained from historical data and deployed in dynamic environments are at risk from data drift. Machine learning models using data collected by sensors making continuous measurements could be impacted by both changes in the device itself and their users, driving drift and impacting safety. To maintain predictive performance, algorithms must be continuously monitored and tuned to overcome fundamental changes to both input data (covariate shift) and the relationship with the output (concept drift). Here, we aim to understand how changes to user behaviour, physiology and sensors could impact the safety of models using automated sensor readings from continuous glucose monitors (CGM).

**Methods and analysis:**

In this paper, we investigate how data drift in a machine learning model trained to predict short-term risk from blood glucose control for individuals with type-1 diabetes. We simulate how changes in both user behaviour and accuracy of the sensor could lead to covariate shift and concept drift. For each scenario, we quantify the changes to input data (Jensen-Shannon divergence), the impact to model performance metrics and the explainability of the model (ie, shift in feature importance).

**Results:**

We demonstrate that using a combination of covariate shift detection, multiple performance metrics and feature importance offers a powerful methodology of identifying different types of drifts in sensor data. For blood glucose management, our scenarios focused on user behaviour (ie, changes to blood glucose dynamics and CGM use) and device/sensor noise and variability, finding more simplistic approaches to drift detection could incorrectly identify risk to model safety.

**Conclusion:**

Machine learning and AI can enhance clinical decision-making, but often lack the transparency required to ensure ongoing safety. Combining complementary monitoring techniques enables clearer identification of changes in data or model behaviour, helping determine when retraining or intervention is needed.

**WHAT IS ALREADY KNOWN ON THIS TOPIC**

- Machine learning (ML) models are increasingly used in healthcare to interpret real-time physiological data and predict short-term clinical risk.
- In diabetes care, for example, algorithms trained on continuous glucose monitoring data can predict hypoglycaemia and hyperglycaemia and support insulin delivery.
- However, these models may become unsafe when underlying data distributions change due to shifts in patient physiology, behaviour, device performance or clinical practice. Such changes, known as data drift, can affect both the input data (covariate shift) and the input–output relationship (concept drift). Effective monitoring strategies are needed to ensure model safety and interpretability in these evolving environments.

**WHAT THIS STUDY ADDS**

- We show that combining complementary monitoring approaches: (1) statistical detection of data distribution changes (eg, Jensen-Shannon distance), (2) ongoing evaluation of model performance and (3) explainable AI methods to track how the model uses its inputs provides deeper insight into model reliability and emerging safety risks.

**HOW THIS STUDY MIGHT AFFECT RESEARCH, PRACTICE OR POLICY**

- ML and artificial intelligence (AI) can enhance patient monitoring and decision support, but often lack the transparency needed for effective oversight. The proposed monitoring framework can identify when a model may require retraining, distinguish between changes in the patient population versus sensor performance and support safer, more interpretable AI integration both in diabetes care and across other domains of digital health.

## Introduction

Wearable devices are now common to help self-management of chronic diseases. Individuals with type-1 diabetes (T1D) can use continuous glucose monitors (CGM) to provide real-time data on their blood glucose (BG) levels. T1D is a chronic metabolic disorder where the body produces little or no insulin, meaning external insulin is needed for BG control. People with T1D face a daily balance to control their BG and minimise risk from acute and long-term severe complications. Predictive algorithms, including machine learning (ML), can use CGM data to predict short-term risk from hypoglycaemia and hyperglycaemia, alert users and support insulin delivery. Recent work has included developing ML models for T1D which have interpretable features,^1^ explainable predictions^2^ and can be understood by laypeople.^3^ These factors are not only important for safe ML but also help reduce longer-term risk for users by offering insight into their BG control. For example, aggregated feature importance of ML models can highlight risk factors and provide users with actionable feedback to improve future control. However, both the safety and interpretability of these models can be negatively impacted over time.^4^

In general, the predictive power of ML algorithms degrades over time. Fundamentally, most predictive models are developed and trained using historical data, meaning that changes in their deployed operational circumstances may risk their efficacy and safety. These changes, referred to as data drift,^5 6^ can be categorised into a systematic shift in the underlying distribution of the input data or features P(X) (ie, covariate shift: P_t_(X)≠P_t+dt_(X) where t is training time), target variables P(Y) (ie, prior probability shift: P_t_(Y)≠P_t+dt_(Y)) or a change in the statistical relationship between input features and target variables (ie, concept drift: P_t_(Y|X)≠P_t+dt_(Y|X)). Generalisation aims to make ML models robust to unseen data (eg, preprocessing and normalisation techniques^7–9^); however, this does not typically consider unseen data to be drawn from a different distribution from training or concept drift. Therefore, ML models must be monitored to ensure predictive performance and the overall safety of the system.^10 11^ Previous work has considered monitoring drift through univariate statistical tests of both covariates and the target,^12 13^ tracking model performance metrics^14 15^ and post hoc model explanations.^16 17^ However, there is no unified framework to identify different types of drift (covariate, concept, target) and understand suitable points to retrain or replace deployed models.

ML models must be both safe and interpretable to comply with medical governance.^18 19^ Explainability of ML models (coined XAI) has become central to deployed systems, both to attribute decision making on a patient-by-patient basis^19^ and monitor ongoing safety.^20^ Recent work addressing data drift in medicine includes using XAI to track the prediction of sepsis,^17^ emergency department admissions,^16^ tracking calibration drift to monitor predictions of mortality^21^ and tracking discrimination power and underlying changes in data distributions for clinical prediction models.^22^ For models using automated sensor readings, such as CGM, it is critical to understand how changes to user behaviour, physiology and increased sensor noise could impact the safety of the system.

In this paper, we investigate how data drift could impact the safety of a ML model trained to predict short-term risk from BG control (ie, hypoglycaemia) for individuals with T1D. We consider real-world scenarios such as changes to user behaviour (ie, choices made daily about BG control and sensor usage) and the sensor (eg, degradation in sensor accuracy, systematic differences between sensors). For each scenario, we quantify data drift (covariate shift, concept drift, prior probability shift) through statistical analysis of covariates and targets, tracking of model performance metrics and changes to feature importance from explanation techniques.

The key aims are (1) to demonstrate a holistic approach to drift detection and (2) to understand how common real-world scenarios for BG control affect the safety of prediction. We first introduce the methodology including the Jensen-Shannon (JS) divergence. We then present the baseline ML model along with results of our scenarios for user behaviour (subsections ‘Drift due to stress or behavioural change’, ‘Drift due to lower CGM use’) and sensors (subsections ‘Device and sensor changes’, ‘Increased sensor or device noise’). We then discuss the practical implications, before concluding.

## Methods

### Data

We develop an ML model to predict the short-term risk from hypoglycaemia using real-world data collected by CGM devices. We use data from ‘A Randomized Trial Comparing Continuous Glucose Monitoring With and Without Routine Blood Glucose Monitoring in Adults with Type 1 Diabetes’ (REPLACE).^23^ The study recruited 225 adults with T1D (duration >12 months) who are using an insulin pump, have haemoglobin A1c (HbA1c) level ≤ 9.0% and have demonstrated previous control and awareness over their BG. For each user, approximately 6 months’ worth of CGM data (one reading every 5 min) is collected using the Dexcom G4 Platinum CGM. REPLACE represents a generalised cohort with 49.8% being female and an average age of 44±14 years, with 47% being prior CGM users. More detailed information can be found in Aleppo *et al*, 2017.^23^ The Dexcom G4 Platinum represents an earlier generation of devices; however, the relatively high noise/error rate compared with its successors is useful in this context for emulating potential variations and drift.^24^

### Feature generation 

We generate a total of 29 features from raw CGM data, given in online supplemental Table 1. We directly build on prior work developing interpretable features, while retaining predictive accuracy.^1 2^ We summarise glucose control into short-term (1 hour), medium-term (1 day) and long-term (1 week) baselines. We generate a set of features for each unique CGM reading. For each timespan, we quantify the variability in CGM readings using rate of change calculated as the average difference between readings over a given baseline.

10.1136/bmjdhai-2025-000269.supp1 Supplementary data

For training, we filter out data where an individual’s CGM usage is less than 80% over the past week before the current CGM reading. The effects of lower usage are explored in subsection ‘Drift due to lower CGM use’.

### Target

We aim to identify future short-term risk due to low BG (ie, hypoglycaemia, <70 mg/dL) occurring up to 60 min in advance of the current CGM reading. This target is binarised to denote if an event will occur in the next hour.

### Modelling

Gradient boosted tree-based models are a flexible and powerful methodology for generating predictions from tabular data. However, the usual choice for predicting future BG readings (and hence risk) is time-series based approaches (eg, Recurrent neural networks, long short-term memory).^25^ While offering predictive performance, they lack interpretability. Recent work^1 2^ introduced the notion of using feature-based techniques to predict BG, offering explainability through the utilisation of decision-tree based models. We employ these through the XGBoost framework,^26 27^ splitting the CGM data into a training set (75%) and hold-out test set (25%) grouped at the patient level. Model performance was evaluated using the area under the receiver operating characteristic curve (AUROC) and average precision (AP), along with fixed measures of specificity and sensitivity. Additional information is given in online supplemental Matrials S1.

### Model explainability

The relative importance of each feature for predicting hypoglycaemia risk is found by using the TreeExplainer algorithm implemented in the SHAP (SHapley Additive exPlanations) library.^19 20^ SHAP offers local explanations (ie, for individual predictions) and aggregates consistently enabling global explanations (ie, per CGM user/for a given timespan/overall). Explainability provides immediate insight to individuals using CGM and their corresponding clinical care teams.^2^

### Detecting covariate shift

To detect covariate shift, we need to quantify the difference between distributions of variable size (ie, of the training set and the current period of operation). Common choices for measuring the difference between two probability distributions are the Kullback-Leibler (KL) divergence^28^ and the JS divergence.^29^ Despite the KL’s popularity in drift detection^30^, it has weaknesses such as asymmetry and that it can extend to infinite values when there are 0 probability events.^31^ The JS divergence solves these weaknesses by considering the mutual information between the mixture distributions giving it a finite range between 0 (ie, where the distributions are exactly the same) and 1 (completely different).

To quantify covariate shift, we first calculate the JS divergence between the probability distributions of the training data and the period of interest (test set), for every feature. Despite the JS divergence generalising to higher dimensionality (ie, full covariate space could be considered as one multivariate distribution), this enables individual insight into which features are driving potential covariate shift. We then take the simple mean of the JS divergence across all covariates to summarise.

Despite this, the magnitude of the JS divergence can vary with the number of bins of the covariate’s probability distribution.^32 33^ While prior work has defined a suitable number of bins based off the sample size of the original distribution alone^34^, when including covariates with predefined number of bins (eg, categorical variables) consistency cannot be ensured. To account for this, we also define a normalised JS divergence by considering the number of standard deviations ( σ ) between the test set’s JS divergence value and the mean of 1000 bootstrapped samples (of equal size) drawn from the training set. By definition, the bootstrap samples are consistent with the training set (ie, $JS(P\;\|\;Q)\to 0\;\text{as}n_{\text{samples}}\to \infty \;width_{bin}\to 0$ ) therefore, the distance characterises the covariate specific noise. Higher positive σ  values indicate greater difference and hence covariate shift, which can be directly compared between covariates and different test sets. We call this value the bootstrap normalised JS divergence and describe its calculation in more detail in online supplemental Materials S2. Since we do not explicitly independently validate the normalised JS divergence here, we identify drift using the (unnormalised) JS divergence. However, the normalised version is presented throughout to investigate the effect of sample size on drift detection.

### Patient and public involvement

Patients and public were involved in the design of the study through the identification of usage scenarios which could lead to problems with ML supporting BG management. This information was collected as part of the Codesigning Trustworthy Autonomous Diabetes Systems project and outcomes of sessions are published here.^3^

## Results

Here we describe the baseline performance of our ML model predicting hypoglycaemia (Section 3.1) before presenting how user behaviour/physiology (Section 3.2), device use (Section 3.3), sensor variation (Section 3.4) and degradation (Section 3.5) could cause drift.

### Baseline model

#### Performance

**Figure 1:**
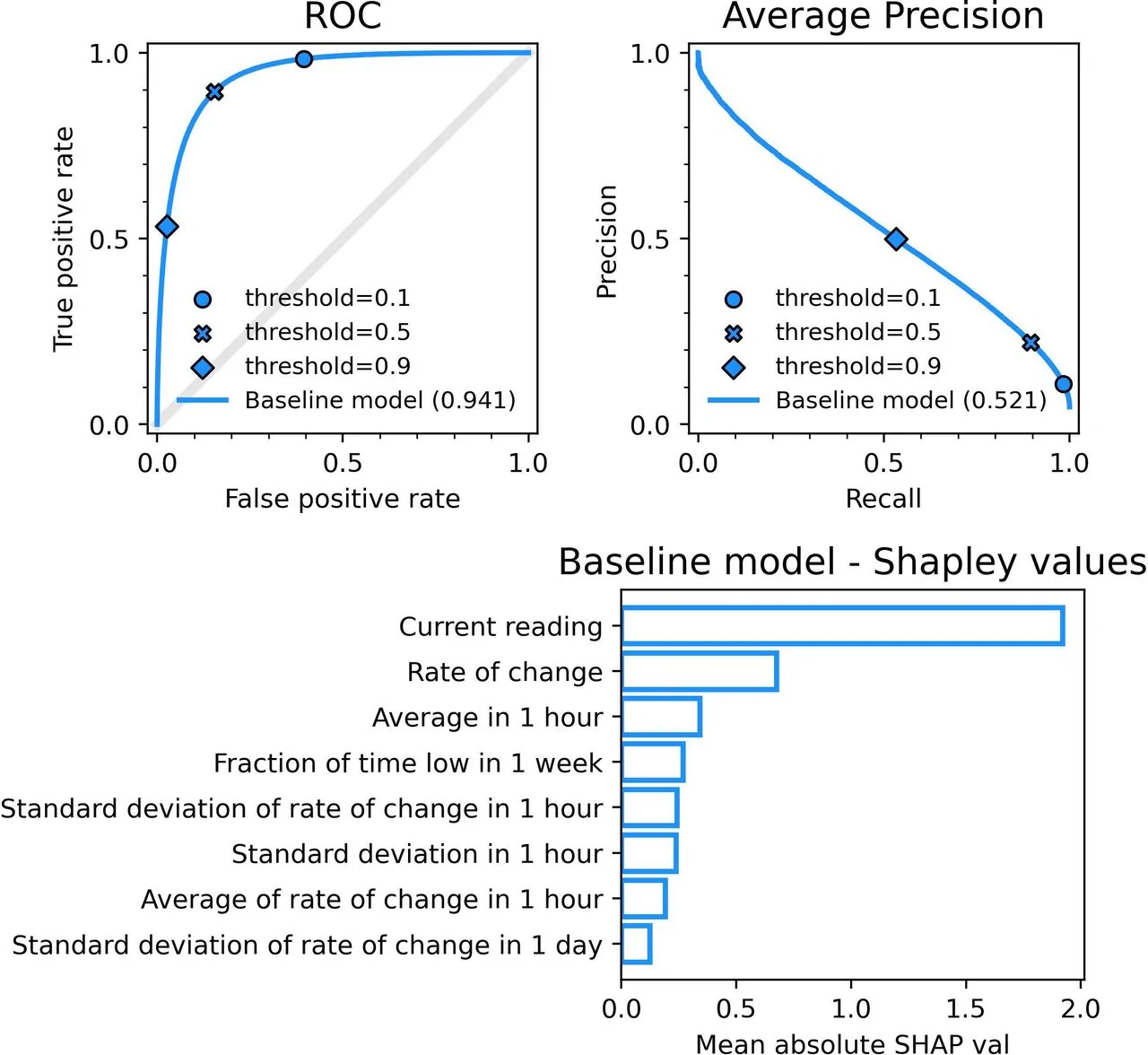
ROC (top-left) and precision-recall (top-right) for our model. A zero-skill model is represented by the grey line in the ROC panel. The total area under each curve (ie, AUROC or AP) is given in the brackets. (Bottom) SHAP importance for the top eight features. AP, average precision; AUROC, area under the receiver operating characteristic curve; ROC, receiver operating characteristic; SHAP, SHapley Additive exPlanations.

Figure 1 displays the ROC (top-left) and precision-recall (top-right) curves of our hypoglycaemia model, evaluated on the hold-out test set. A high AUROC value is obtained (0.946). We overplot the true and false positive rates by dichotomising at probability thresholds of 0.1 (circle), 0.5 (cross) and 0.9 (diamond). The low skewness of the markers on the ROC curve indicates the model is well balanced to classify positive and negative classes. We note a lower AP value (0.521). Since $Precision=\frac{TP}{TP+FP}$ , even if the classifier has a low false positive rate, AP is more sensitive to the positive class (hypoglycaemic; 4.68% of samples) which is outnumbered by the negative class (normal glycaemia and hyperglycaemic; 95.32%). Dichotomising our predictions at a probability threshold of 0.5, our model returns a sensitivity of 0.896 and specificity of 0.845.

#### Explainability

We use SHAP to evaluate the most important features for the prediction of hypoglycaemia. Figure 1 (bottom panel) presents the mean absolute SHAP value for the eight most important features. The two instantaneous features ‘current reading’ (most recent CGM reading) and ‘rate of change’ (current rate of change of CGM readings per minute) dominate in feature importance. This demonstrates that predictions primarily leverage short-term data and longer-baseline features only provide second-order corrections for prediction. In online supplemental Materials Figure 1, we show how the distribution of feature values correlates with individual SHAP values.

### Drift due to stress or behavioural change

#### Data

Here, we investigate how changes in BG dynamics can cause drift. Patterns in BG control can experience variation due to factors such as education,^35^ physiology,^36^ stress^37^ and activity levels.^38^ For example, stress releases hormones such as cortisol and epinephrine which provide insulin resistance, preventing energy absorption, driving BG levels higher and variability.^39^ Individuals also make specific choices with their glycaemic control to mitigate risk. An example would be to ‘ride high’, where an individual, in anticipation of a busy day where less attention can be paid to their glycaemic control, will intentionally overload in carbohydrate intake to avoid more immediate short-term risk from hypoglycaemia.

To characterise changes in BG dynamics and its impact on the prediction of hypoglycaemia, we classify our test data into four categories (‘low risk range’, 'below range’, ‘above range’ and ‘high variance’). The remaining test data are labelled unclassified. These patterns are identified over 3-day periods according to the criteria listed within table 1. We require CGM usage to be greater than 80% when defining these categories. While our categories aim to capture broad patterns in glucose dynamics, they don’t strictly align with clinical guidelines (eg, ‘high variance’ in glycaemic variability often corresponds to >36% coefficient of variance^40^). Our classifications strike a balance between data sample size and alignment with clinical definitions.

**Table 1:** CGM data characterised and grouped by distinct patterns in BG dynamics

Pattern	Description	Selection (over the past 3 days)			Proportion of test set
		Criterion 1	Criterion 2	Criterion 3	
‘Low risk range’	BG levels are generally within a range that has low risk for hypoglycaemia and hyperglycaemia and has relatively low variability.	Mean CGM reading 100–140 mg/dL	SD of reading <50 mg/dL		32.1%
‘Below range’	BG levels are generally below the low-risk range, just above the hypoglycaemic threshold for most of the duration. This pattern is likely to include periods of hypoglycaemia and has small variance.	Mean CGM reading <100 mg/dL	SD of reading <50 mg/dL	--	2.5%
‘Above range’	BG levels are generally above the low-risk range. This pattern is likely to include frequent periods of hyperglycaemia. This is designed to emulate ‘riding high’ or other reasons which drive high BG.	Mean CGM reading >140 mg/dL	SD of reading <50 mg/dL	--	28.5%
‘High variance’	BG levels are highly variable, likely to include periods of both frequent hypoglycaemia and hyperglycaemia.	--	SD of reading >80 mg/dL	SD of rate of change >1.2 mg/dL/minute	9.9%
‘Unclassified’	Test data which does not strictly fit into any of the defined categories.	--	--	--	27.1%

Selection criteria and fraction (of test set) are given for each pattern.

BG, blood glucose; CGM, continuous glucose monitoring.

### Covariate shift

Table 2 quantifies drift for each pattern using the mean JS divergence and a bootstrap normalised version. The ‘above range’ had the lowest normalised JS divergence, representing that users most commonly fall here. ‘High variance’ represents the largest shift from typical BG dynamics. We note that the uncorrected JS divergence values correlate with class size, highlighting the importance of normalising by sample size. This is seen most notably for ‘below range’ which has a high JS divergence, but after correction had a more modest drift (bootstrap normalised JS divergence).

**Table 2:** Mean JS divergence and bootstrap normalised JS divergence (ie, number of SD *(σ)* from bootstrapped distribution of JS values) for identified BG dynamic pattern

	Low risk range	Below range	Above range	High variance	Unclassified
Mean JS divergence	0.0154	0.1092	0.0091	0.0419	0.0181
Mean bootstrap normalised JS divergence ( σ )	27.0	21.65	9.71	39.52	18.69

BG, blood glucose; JS, Jensen-Shannon.

#### Predictive performance

**Figure 2:**
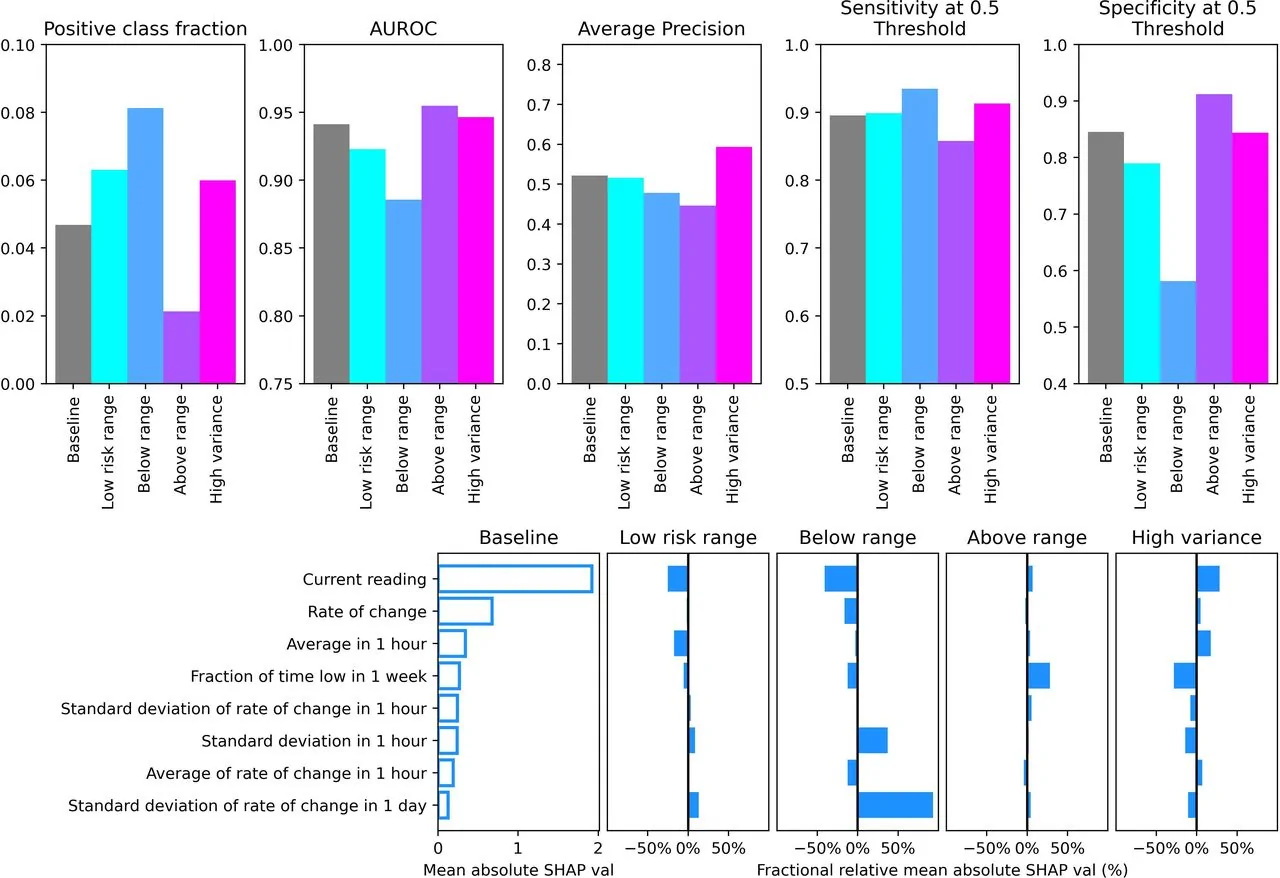
(Top) Summary of model performance for each characterised BG control pattern (and baseline); (left-to-right) positive class fraction, AUROC, average precision, sensitivity and specificity (dichotomised at 0.5 probability). (Bottom) Feature importance as measured by SHAP. The left-most panel shows the baseline importance with all other panels showing the relative change for a particular pattern of behaviour. AUROC, area under the receiver operating characteristic curve; BG, blood glucose; SHAP, SHapley Additive exPlanations.

Figure 2 (top row) shows the positive class fraction (target) and performance of the predictive model for each pattern. We choose AUROC, AP, sensitivity/specificity (dichotomised at probability=0.5) as commonly adopted metrics. We find that ‘above range’ leads to an increase in AUROC, while AP decreases. This is mirrored by a drop in sensitivity, with specificity increasing. The opposite trends are found for ‘below range’. Contributing to these changes is class balance and the local performance of our model as data drifts. For example, the ‘below range’ pattern has a higher proportion of hypoglycaemic events but results in a lower true negative rate (specificity) as the model appears to over-predict hypoglycaemia for low readings (but with lower variability). This highlights the danger of using performance metrics alone to identify drift.

Notably, ‘high variance’ has a very high JS divergence despite leading to an increase in predictive performance (AP and sensitivity increase while AUROC and specificity remain consistent). This contrasts with ‘below range’, indicating our model is better skilled at predicting hypoglycaemia resulting from sudden drops in BG.

#### Explanations

Figure 2 (bottom) shows the changes in feature importance for the identified patterns relative to their baseline (left-hand panel). For ‘below range’, the importance of the current reading drops, with the model becoming more reliant on the degree of both short-term and long-term variability. Comparatively, ‘high variance’ uses the current reading and its short-term average to generate predictions. For the ‘low risk range’, we see relatively small changes to the original test set, in parallel with smaller changes to performance.

Overall, this helps provide additional context to both changes in performance and covariate shift. For example, we can understand that a user who is ‘below range’ will have a reasonable detectable covariate shift (JS divergence) and significant drop in performance (AUROC). However, the AP change is minimal and in practice, this may result in a higher false-alarm rate. SHAP helps us understand that our model is less reliant on ‘current reading’ and more reliant on features looking at variability (ie, SD over the last day and hour prior to the hypo event). Physiologically, these features make sense as clinical risk markers for individuals with more recent lower readings and high variability who will generally be at higher risk of hypoglycaemia.

### Drift due to lower CGM use

#### Data

Here, we investigate how CGM use (eg, due to user choice, equipment failure or issues accessing CGM supplies) could cause drift. Our model is trained only on samples where the CGM usage fraction is >0.8 over the prior week. This ensures that features calculated over a 1-week span will be complete. Practically, this limits the use of the model to when CGM use is high, unless the effect of lower use is understood and characterised. We note that a pragmatic approach would be to include lower use in the training sample; however, we aim to characterise how our methodology could detect previously unseen CGM usage patterns. We split our unseen test CGM data into 8 subsets of 0.1 increments of usage fraction over the prior week (up to 0.8).

#### Covariate shift

Table 3 shows the covariate shift measured by JS distances for each subset. Covariate shift increases significantly with decreasing CGM usage fractions.

**Table 3:** Mean JS divergence and bootstrap normalised JS divergence (ie, number of SD *(σ)* from bootstrapped distribution of JS values) for different CGM usage

CGM usage fraction	0.0–0.1	0.1–0.2	0.2–0.3	0.3–0.4	0.4–0.5	0.5–0.6	0.6–0.7	0.7–0.8
Mean JS divergence	0.0838	0.0532	0.0236	0.0192	0.0179	0.0163	0.0148	0.0119
Mean bootstrap normalised JS divergence ( σ )	113.17	48.71	25.46	18.46	19.06	16.61	17.6	12.44

CGM, continuous glucose monitoring; JS, Jensen-Shannon.

#### Predictive performance

**Figure 3:**
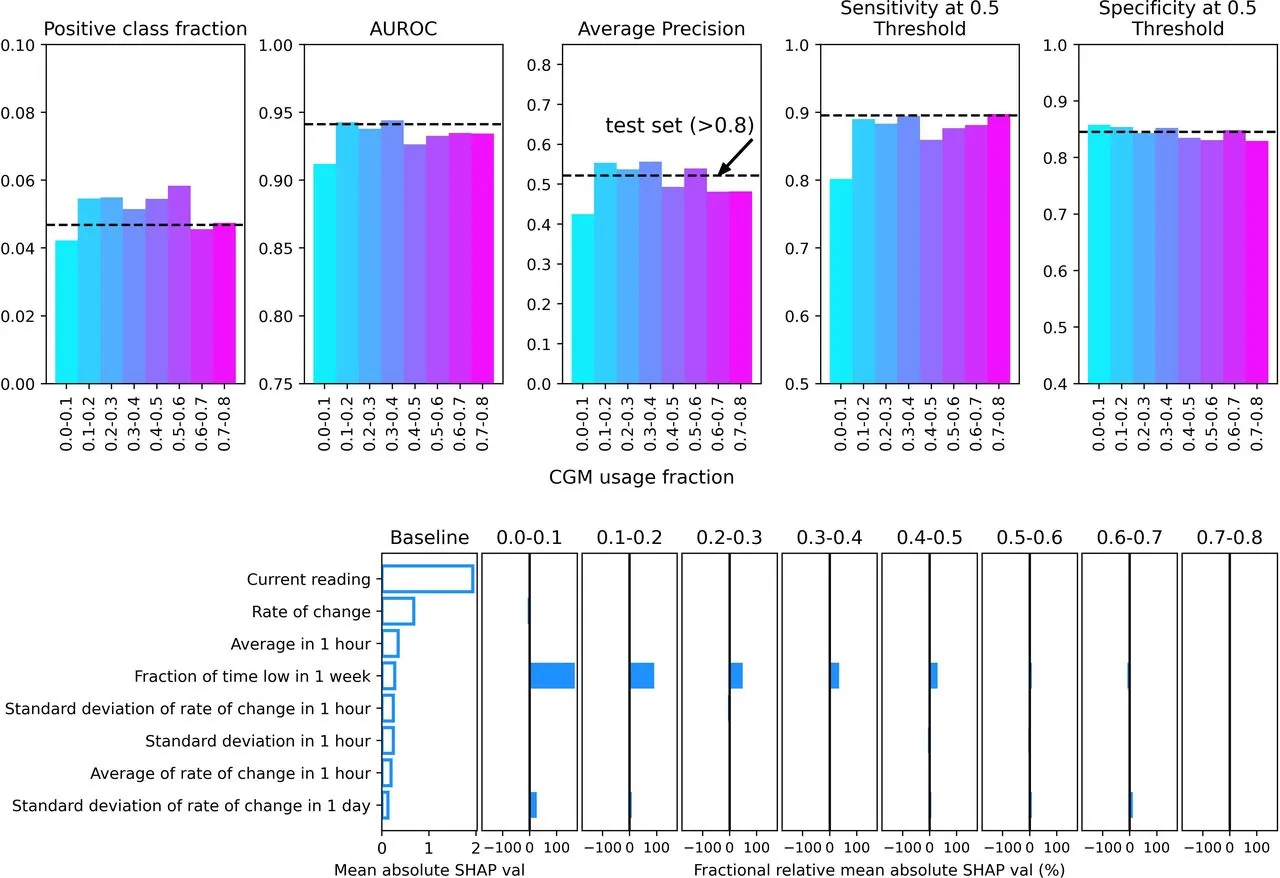
(Top) Summary of model performance for each CGM usage fraction; (left-to-right) positive class fraction, AUROC, average precision, sensitivity and specificity (dichotomised at 0.5 probability). The dashed black line shows the baseline value for the unadjusted test set which considers all data with usage fractions >0.8. (Bottom) Feature importance for prediction as measured by SHAP. The left-most panel shows the baseline importance with all other panels showing the relative change. AUROC, area under the receiver operating characteristic curve; CGM, continuous glucose monitoring; SHAP, SHapley Additive exPlanations.

Figure 3 (top) shows the performance of our model for each CGM usage subset. We find no clear trend for any of our performance metrics. Only the lowest usage fraction (<0.1) results in a significant decrease in model performance.

#### Explanations

Figure 3 (bottom) shows the change in feature importance for different CGM usage fractions. The importance of features computed on a 1-week baseline increases as CGM usage fraction declines, as values fall out of range. Notably, the two instantaneous features ‘current reading’ and ‘rate of change’ maintain their importance for predicting hypoglycaemia risk across CGM usage fractions. Therefore, predictive performance is only marginally affected as CGM use drops, despite a significant drift in JS divergence.

### Device and sensor changes

#### Data

We now consider how a change in sensor or device could cause drift. The typical lifespan of a CGM sensor is 10–14 days (7 days for those used in this dataset). Users will frequently change sensors which have natural fluctuations in recording accuracy due to manufacturing and application. Additionally, there are multiple brands of CGM device on the market, with each brand having multiple generations of devices. Historically, variations across both device and sensor can offset CGM readings by up to 50 mg/dL and it is important to understand how predictive models generalise.^41 42^ Despite recent sensors being factory calibrated more precisely, accuracy may be impacted by inflammatory responses to sensor insertion (ie, until the sensor is immersed in interstitial tissue without inflammation or scarred tissue).^43^ This usually results in a drop in sensitivity and a transient negative offset which sensor algorithms may not characterise.

To emulate the potential effects of changing CGM device or sensor, we apply uniform shifts up or down in CGM readings. A change of device or sensor may also increase noise (eg, sensor offsets or tissue trauma in the immediate vicinity), which is considered independently in subsection ‘Increased sensor or device noise’. We sample 100 000 datapoints from the test set, apply a uniform shift of −20 to –5,+5 and +20 mg/dL to readings and re-generate our features. We keep the original unshifted targets as ‘ground truth’ (ie, classification of hypoglycaemia) in order to consider how these inaccuracies lead to drift and risk to user.

#### Covariate shift

In table 4, we show the mean JS divergence and bootstrap normalised JS divergence of each uniformly shifted test set. We find that the more significant the uniform change, the larger the detected covariate shift.

**Table 4:** Mean JS divergence and bootstrap normalised JS divergence (ie, number of SD *(σ)* from bootstrapped distribution of JS values) for uniform shifts to CGM readings

	−20 mg/dL	−5 mg/dL	0 mg/dL	+5 mg/dL	+20 mg/dL
Mean JS divergence	0.0178	0.0030	0.0017	0.0034	0.0201
Mean bootstrap normalised JS divergence ( σ )	17.95	0.14	−1.49	0.60	21.11

CGM, continuous glucose monitoring; JS, Jensen-Shannon.

#### Predictive performance

**Figure 4:**
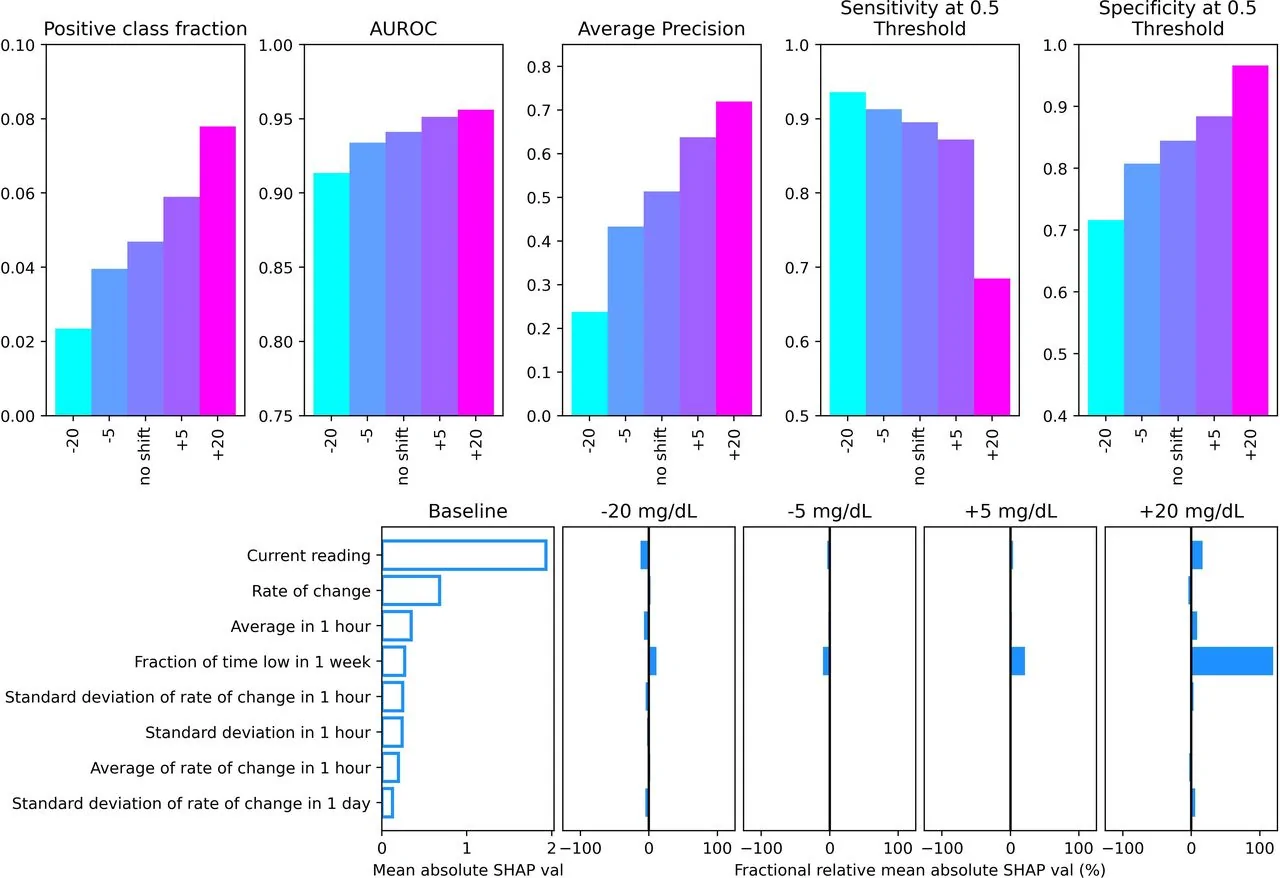
(Top) Summary of model performance for uniform shifts to CGM readings; (left-to-right) positive class fraction, AUROC, average precision, sensitivity and specificity (dichotomised at 0.5 probability). No shift shows the baseline value for the unadjusted test set. (Bottom) Feature importance for prediction as measured by SHAP. The left-most panel shows the baseline importance with all other panels showing the relative change. AUROC, area under the receiver operating characteristic curve; CGM, continuous glucose monitoring; SHAP, SHapley Additive exPlanations.

Figure 4 (top) shows how performance changes with uniform shifts to readings. At shifts of+5/–5 mg/dL, a variation of ≲0.1 is introduced in AP, sensitivity and specificity, indicating only a mild impact to model performance. Increasing to –20/+20 mg/dL, we note significant changes. Again, we note the relationship between positive class fraction and the disparate impact to sensitivity and specificity. This underlines the importance of using multiple methods and metrics to track drift and hence model safety. In particular, +20 mg/dL shows no notable drop in AUROC or AP; however, a significant drop in sensitivity.

While we keep the target fixed, the change in positive class fraction is due to variations in the proportion of current readings falling below 70 mg/dL. No predictions are made while an individual is already in hypoglycaemic range.

#### Explanations

In figure 4 (bottom), we show the changes in feature importance. There are no obvious trends, however, ‘fraction of time low in 1 week’ showed a significant increase in importance for +20 mg/dL due to values falling out of range.

### Increased sensor or device noise

#### Data

We now further explore how variation between sensors and devices could cause drift. We explicitly consider the addition of stochastic noise to CGM readings, rather than a systematic change in readings. As described above, sensors and devices have varying degrees of accuracy and error rate which can change over their lifetime. We perturb CGM readings from the test set with Gaussian random uncorrelated noise, in increments σ=[5%, 10%, 20%] of the measured CGM readings. The features are then re-generated after perturbation. In online supplemental Materials S4, we also explore how sensor degradation over time could cause drift by considering model performance as a function of sensor age (online supplemental Figure 2) shows performance and explanations as in other scenarios, whereas online supplemental Table 2 shows JS divergence).

#### Covariate shift

Table 5 shows the JS divergence and bootstrap normalised JS divergence for increasing degrees of noise. Even a modest addition of 5% noise leads to a very significant covariate shift. Despite added noise resulting in little change in the overall distribution of CGM readings, the scatter of features such as ‘rate of change’ is greatly increased. It is important to not only consider the raw distribution of data but features explicitly when considering covariate shift.

**Table 5:** Mean JS divergence and bootstrap normalised JS divergence (ie, number of sigma relative to bootstrapping) for test sets with increasing degrees of added gaussian noise

	No added noise	5%	10%	20%
Mean JS divergence	0.00275	0.144	0.209	0.251
Mean bootstrap normalised JS divergence ( σ )	−0.21	172.18	257.59	287.23

JS, Jensen-Shannon.

#### Predictive performance

**Figure 5:**
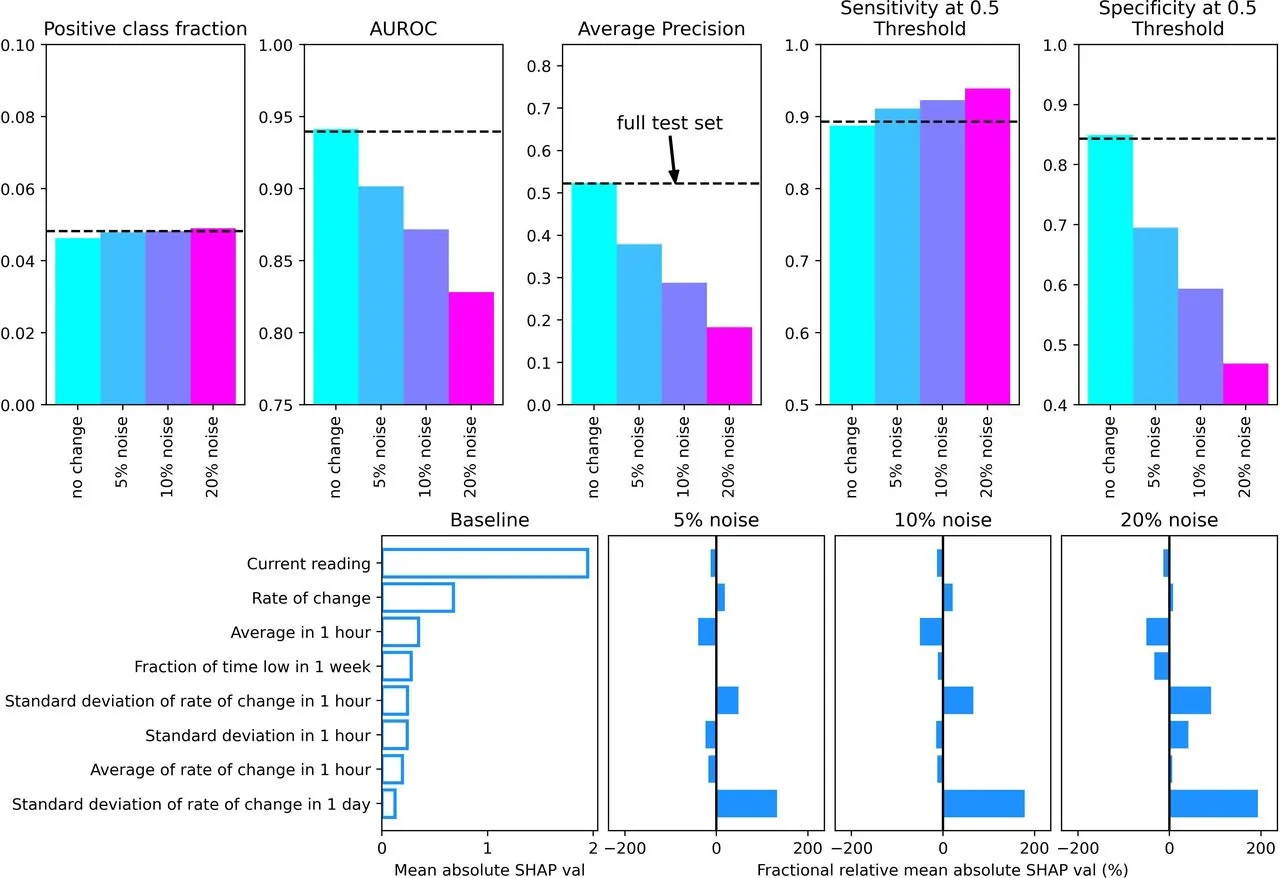
(Top) Summary of model performance for increasing magnitude of added Gaussian noise; (left-to-right) positive class fraction, AUROC, average precision, sensitivity and specificity (dichotomised at 0.5 probability). The dashed black line shows the baseline value for the unadjusted test set. (Bottom) Feature importance for prediction as measured by SHAP. The left-most panel shows the baseline importance with all other panels showing the relative change. AUROC, area under the receiver operating characteristic curve; SHAP, SHapley Additive exPlanations.

Figure 5 (top) shows how model performance changes as an increasing amount of noise is added. The horizontal dashed lines mark the original performance of the test set for comparison. Any addition of noise significantly lowers model performance as marked by AUROC, AP and sensitivity. We note the increase in sensitivity of our model as added noise increases. Since specificity sharply declines, this suggests the drift leads our model to overpredict hypoglycaemia. This is driven by inflated magnitudes of ‘average rate of change’ and related features which quantify rate of change over increasing timescales.

#### Explanations

Figure 5 (bottom) shows how feature importance changes with increasing amounts of noise. There is a strong correlation with the amplitude of added noise and the importance of ‘SD of rate of change’ features. On visual inspection (see online supplemental Figure 1), we note that a sharp drop in BG leads to a significant increase in probability of hypoglycaemia according to our model. An increased magnitude of noise increases the number of perceived sharp drops in BG, hence leading to the overprediction of hypoglycaemia (and drop in specificity). The observation that even a low 5% additional noise results in significant drops in false recall suggests that our model is very sensitive to the inflated amplitudes of rate of change. Online supplemental Materials S3 provides more insight into the relationship between the distributions of feature values and SHAP values.

## Discussion

We explored how changes in user behaviour or sensor accuracy could lead to data drift and ultimately affect the safety of a model predicting hypoglycaemia. These scenarios could be easily misinterpreted by only considering the input data, a single model performance metric or feature importance in isolation when trying to detect drift.

We introduced a framework to monitor and understand drift. First, the JS divergence, and a bootstrap normalised version, enables consistent comparisons of drift between test sets of different sample sizes. This facilitates covariate shift to be evaluated at variable intervals. Second, we show that using multiple performance metrics is critical to detect drift due to disparate impact on positive and negative classes, which one metric alone may not be sensitive to. Finally, quantifying the change in feature importance using SHAP enables intuition on how the model should be made more robust once drift is detected.

For user behaviour, users who are ‘above range’ (3.2) despite only having a moderate covariate shift relative to baseline and no negative change in AUROC are at elevated risk of false negatives (ie, hypoglycaemia missed by the model). This risk can only be identified by considering other performance metrics (ie, AP, sensitivity) and explained by a decreased model reliance on rate of change features. Conversely, users who are ‘below range’ may be identified with a reasonable covariate shift and a drop in AUROC and specificity; however, the model’s sensitivity remains high, which manifests as a higher false-alarm rate. Different subselections of behaviours contained within the training set represent examples of data drift, rather than concept drift.

For device and sensor changes, adding noise results in large covariate shifts, significant performance degradation and a change in the importance of rate of change (of CGM readings). Our scenarios adding both systematic (3.4) and stochastic (3.5) noise are explicitly concept drift (ie, retraining needed), since we alter features while keeping the target fixed. In general, concept drift is detected through a combination of covariate shift (JS divergence), changes to various model performance metrics and changes to feature importance. Dichotomising at a fixed threshold and explicitly considering sensitivity and specificity may be most effective for detecting drift in this context. This helps unpack the relationship between sensitivity, specificity and changes in class balance (ie, target drift).

In online supplemental Materials S5, we consider how our framework could be practically adopted for users of CGM. While our study isolates drift for specific scenarios, in practice data may contain multiple effects (ie, both user and sensor) which are hard to deconvolve. We consider how covariates, performance and explanations of our model drift up to 150 days after an initial training period (online supplemental Figure 4). We find a gradual, although small, decline in model performance (online supplemental Figure 3), underlined by changes to the importance of longer-term features.

For CGM users and healthcare professionals, this framework offers greater safety and insight into ML models using CGM readings and for BG risk predictions overall. Data drift can result from both technology and an individual’s BG dynamics, which both need to be considered individually when considering risk factors and decision making (eg, around dosing). We look to future work to consider how our framework can be applied to understand and identify when models should be retrained and how it can be used to isolate technology ‘noise’ and changes to behaviour or BG dynamics.

Limitations of this study include the simulated and idealistic nature of our experiments. While providing a practical guide on isolated data drift and concept drift in CGM prediction, real-world CGM use may incorporate a combination of variable noise and user behaviours which could be hard to isolate. In online supplemental Materials S5, we conduct a different experiment to consider how a CGM user’s data would drift month-by-month to better understand appropriate points for retraining. Other limitations include the dataset, which utilises prior generation CGM devices.

## Conclusion

We presented a series of scenarios intended to demonstrate how data drift and concept drift could impact ML models predicting hypoglycaemia for individuals with T1D. Our scenarios focused on user behaviour (ie, changes to BG dynamics or CGM use) and device/sensor noise and variability. We demonstrated a framework to monitor and understand drift using (1) the JS divergence to characterise changes to feature values, (2) a variety of performance metrics and (3) tracked changes in feature importance as measured by SHAP offers a powerful methodology of identifying different types of drift and model safety.

## Data Availability

Data are available in a public, open access repository. Raw CGM data are taken from the publicly available study: 'A Randomized Trial Comparing Continuous Glucose Monitoring With and Without Routine Blood Glucose Monitoring in Adults with Type 1 Diabetes' (REPLACE).^23^ Augmented data (ie, simulation of added noise and errors) is available on reasonable request.

## References

[1] Dave D , DeSalvo DJ , Haridas B , et al . Feature-Based Machine Learning Model for Real-Time Hypoglycemia Prediction. J Diabetes Sci Technol 2021;15:842–55. 10.1177/1932296820922622 32476492 PMC8258517

[2] Duckworth C , Guy MJ , Kumaran A , et al . Explainable Machine Learning for Real-Time Hypoglycemia and Hyperglycemia Prediction and Personalized Control Recommendations. J Diabetes Sci Technol 2024;18:113–23. 10.1177/19322968221103561 35695284 PMC10899844

[3] Ayobi A , Hughes J , Duckworth CJ , et al . Computational notebooks as co-design tools: engaging young adults living with diabetes, family carers, and clinicians with machine learning models. Proceedings of the 2023 CHI Conference on Human Factors in Computing Systems; Hamburg Germany, 2023 Available: https://dl.acm.org/doi/proceedings/10.1145/3544548

[4] Sabatini A , Cenerini C , Vollero L , et al . Calibrating Glucose Sensors at the Edge: A Stress Generation Model for Tiny ML Drift Compensation. BioMedInformatics 2024;4:1519–30. 10.3390/biomedinformatics4020083

[5] Quiñonero-Candela J , et al . Dataset Shift in Machine Learning. Mit Press, 2022.

[6] Moreno-Torres JG , Raeder T , Alaiz-Rodríguez R , et al . A unifying view on dataset shift in classification. Pattern Recognit DAGM 2012;45:521–30. 10.1016/j.patcog.2011.06.019

[7] Dexter GP , et al . Generalization of machine learning approaches to identify notifiable conditions from a statewide health information exchange. AMIA Summits on Translational Science Proceedings; 2020:152. PMC7233074 32477634

[8] Hendrycks D , et al . Augmix: A simple data processing method to improve robustness and uncertainty. arXiv 2019.

[9] Pan X , et al . Proceedings of the european conference on computer vision (ECCV). Two at once: enhancing learning and generalization capacities via ibn-net; 2018

[10] Hoens TR , Polikar R , Chawla NV . Learning from streaming data with concept drift and imbalance: an overview. Prog Artif Intell 2012;1:89–101. 10.1007/s13748-011-0008-0

[11] Mallick A , et al . Matchmaker: Data drift mitigation in machine learning for large-scale systems. Proceedings of Machine Learning and Systems 2022;4:77–94.

[12] Lipton Z , Wang Y-X , Smola A . Detecting and correcting for label shift with black box predictors. International conference on machine learning; PMLR, 2018

[13] Rabanser S , Günnemann S , Lipton Z . Failing loudly: An empirical study of methods for detecting dataset shift. Adv Neural Inf Process Syst 2019;32.

[14] Bayram F , Ahmed BS , Kassler A . From concept drift to model degradation: An overview on performance-aware drift detectors. Knowl Based Syst 2022;245:108632. 10.1016/j.knosys.2022.108632

[15] Gama J , Sebastião R , Rodrigues PP . On evaluating stream learning algorithms. Mach Learn 2013;90:317–46. 10.1007/s10994-012-5320-9

[16] Duckworth C , Chmiel FP , Burns DK , et al . Using explainable machine learning to characterise data drift and detect emergent health risks for emergency department admissions during COVID-19. Sci Rep 2021;11:23017. 10.1038/s41598-021-02481-y 34837021 PMC8626460

[17] Rahmani K , Thapa R , Tsou P , et al . Assessing the effects of data drift on the performance of machine learning models used in clinical sepsis prediction. Int J Med Inform 2023;173:104930. 10.1016/j.ijmedinf.2022.104930 36893656

[18] Cruz Rivera S , Liu X , Chan A-W , et al . Guidelines for clinical trial protocols for interventions involving artificial intelligence: the SPIRIT-AI extension. The Lancet Digital Health 2020;2:e549–60. 10.1016/S2589-7500(20)30219-3 33328049 PMC8212701

[19] Lundberg SM , Erion G , Chen H , et al . From Local Explanations to Global Understanding with Explainable AI for Trees. Nat Mach Intell 2020;2:56–67. 10.1038/s42256-019-0138-9 32607472 PMC7326367

[20] Lundberg SM , Nair B , Vavilala MS , et al . Explainable machine-learning predictions for the prevention of hypoxaemia during surgery. Nat Biomed Eng 2018;2:749–60. 10.1038/s41551-018-0304-0 31001455 PMC6467492

[21] Davis SE , et al . Calibration drift among regression and machine learning models for hospital mortality. AMIA Annual Symposium Proceedings; 2017 10.1093/jamia/ocx030 PMC5977580 29854127

[22] Chi S , Tian Y , Wang F , et al . A novel lifelong machine learning-based method to eliminate calibration drift in clinical prediction models. Artif Intell Med 2022;125:102256. 10.1016/j.artmed.2022.102256 35241261

[23] Aleppo G , Ruedy KJ , Riddlesworth TD , et al . REPLACE-BG: A Randomized Trial Comparing Continuous Glucose Monitoring With and Without Routine Blood Glucose Monitoring in Adults With Well-Controlled Type 1 Diabetes. Diabetes Care 2017;40:538–45. 10.2337/dc16-2482 28209654 PMC5864100

[24] Nakamura K , Balo A . The Accuracy and Efficacy of the Dexcom G4 Platinum Continuous Glucose Monitoring System. J Diabetes Sci Technol 2015;9:1021–6. 10.1177/1932296815577812 25802469 PMC4667335

[25] Oviedo S , Vehí J , Calm R , et al . A review of personalized blood glucose prediction strategies for T1DM patients. Int J Numer Method Biomed Eng 2017;33:e2833. 10.1002/cnm.2833 27644067

[26] Chen T , Guestrin C . Xgboost: a scalable tree boosting system. Proceedings of the 22nd acm sigkdd international conference on knowledge discovery and data mining; 2016

[27] Akiba T , et al . Optuna: a next-generation hyperparameter optimization framework. Proceedings of the 25th ACM SIGKDD international conference on knowledge discovery & data mining; 2019

[28] Kullback S , Leibler RA . On Information and Sufficiency. Ann Math Statist 1951;22:79–86. 10.1214/aoms/1177729694

[29] Lin J . Divergence measures based on the Shannon entropy. IEEE Trans Inform Theory 1991;37:145–51. 10.1109/18.61115

[30] Goldenberg I , Webb GI . Survey of distance measures for quantifying concept drift and shift in numeric data. Knowl Inf Syst 2019;60:591–615. 10.1007/s10115-018-1257-z

[31] Yang W , Su R , Cheng Y , et al . A concept drift detection approach based on jensen-shannon divergence for network traffic classification. Proceedings of the 2022 5th International Conference on Artificial Intelligence and Pattern Recognition; 2022 Available: https://dl.acm.org/doi/proceedings/10.1145/3573942

[32] Bu Y , Zou S , Liang Y , et al . Estimation of KL Divergence: Optimal Minimax Rate. IEEE Trans Inform Theory 2018;64:2648–74. 10.1109/TIT.2018.2805844

[33] Bu Y , Zou S , Liang Y , et al . Estimation of kl divergence between large-alphabet distributions. 2016 IEEE International Symposium on Information Theory (ISIT); IEEE, Barcelona, Spain. 10.1109/ISIT.2016.7541473

[34] Rice JA , Rice JA . Mathematical Statistics and Data Analysis. 371. Thomson/Brooks/Cole Belmont, CA, 2007.

[35] Edraki M , Zarei A , Soltanian M , et al . The Effect of Peer Education on Self-Care Behaviors and the Mean of Glycosylated Hemoglobin in Adolescents with Type 1 Diabetes: A Randomized Controlled Clinical Trial. Int J Community Based Nurs Midwifery 2020;8:209–19. 10.30476/ijcbnm.2020.82296.1051 32656273 PMC7334744

[36] Chiang JL , Kirkman MS , Laffel LMB , et al . Type 1 diabetes through the life span: a position statement of the American Diabetes Association. Diabetes Care 2014;37:2034–54. 10.2337/dc14-1140 24935775 PMC5865481

[37] Buchberger B , Huppertz H , Krabbe L , et al . Symptoms of depression and anxiety in youth with type 1 diabetes: A systematic review and meta-analysis. Psychoneuroendocrinology 2016;70:70–84. 10.1016/j.psyneuen.2016.04.019 27179232

[38] Tonoli C , Heyman E , Roelands B , et al . Effects of different types of acute and chronic (training) exercise on glycaemic control in type 1 diabetes mellitus: a meta-analysis. Sports Med 2012;42:1059–80. 10.1007/BF03262312 23134339

[39] Mosbah AAAE-R , Abd-Ellatif NAB , Sorour EI . Influence of serum cortisol levels on glycemic control in children with type 1 diabetes. J Egypt Soc Parasitol 2011;41:777–84. 22435169

[40] American Diabetes Association . Introduction: Standards of Medical Care in Diabetes—2022. Diabetes Care 2022;45:S1–2. 10.2337/dc22-Sint 34964812

[41] Pleus S , Kamecke U , Waldenmaier D , et al . Time in Specific Glucose Ranges, Glucose Management Indicator, and Glycemic Variability: Impact of Continuous Glucose Monitoring (CGM) System Model and Sensor on CGM Metrics. J Diabetes Sci Technol 2021;15:1104–10. 10.1177/1932296820931825 32513087 PMC8442198

[42] Pleus S , Stuhr A , Link M , et al . Variation of Mean Absolute Relative Differences of Continuous Glucose Monitoring Systems Throughout the Day. J Diabetes Sci Technol 2022;16:649–58. 10.1177/1932296821992373 33615834 PMC9294578

[43] Klonoff DC , Ahn D , Drincic A . Continuous glucose monitoring: A review of the technology and clinical use. Diabetes Res Clin Pract 2017;133:178–92. 10.1016/j.diabres.2017.08.005 28965029

