# Supplementary material for: How data drift impacts the safety and interpretability of machine learning models predicting risk from blood glucose control
Source: BMJ Digit Health Ai. 2026 Feb 9;2(1):e000269. doi: 10.1136/bmjdhai-2025-000269 (PMC13528331; doi:10.1136/bmjdhai-2025-000269)
Supplement: Supplementary file 1 [file bmjdhai-2-1-bmjdhai-2025-000269-s001.pdf]

SUPPLEMENTARY MATERIALS

S1: Additional Methods Information

Feature	Description	Time period (s)
Current reading	Most recent CGM glucose reading	N/A
Rate of change	Average difference between consecutive CGM readings over the last minute	N/A
Device usage fraction	Fraction of time (as specified by the time period) of which the CGM device was used	(One-hour, one-day, one-week)
Fraction of time high	Fraction of time (as specified by the time period) of which CGM readings were above 270 mg/dL	(One-hour, one-day, one-week)
Fraction of time low	Fraction of time (as specified by the time period) of which CGM readings were below 70 mg/dL	(One-hour, one-day, one-week)
Average	Mean of glucose readings within specified time period	(One-hour, one-day, one-week)
Standard deviation	Standard deviation of glucose readings within specified time period	(One-hour, one-day, one-week)
Average of rate of change	Mean of CGM reading rate of change within specified time period	(One-hour, one-day, one-week)
Standard deviation of rate of change	Standard deviation of CGM reading rate of change within specified time period	(One-hour, one-day, one-week)
Maximum of rate of change	Maximum of rate of change values (most rapidly increasing) over specified time period	(One-hour, one-day, one-week)
Minimum of rate of change	Minimum of rate of change values (most rapidly decreasing) over specified time period	(One-hour, one-day, one-week)

Table 1: List of features used to predict hypoglycaemia with our ML model. Where listed, features are computed using multiple lookback time periods to summarise information on different timescales.

In Supplementary Table 1, we show the complete set of features used for modelling. These summarise glucose control over one-hour, one-day and one-week to capture control over different time baselines.

To optimize hyperparameters of our XGBoost models, we use five-fold cross-validation grouped at the patient level using a sampler (Tree-structured Parzen Estimator) implemented with the Optuna library[27]. The non-overlapping patient grouping ensures that no study participant's data lies in both training and validation to avoid overfitting/data leakage. Average precision is chosen as the tuning metric during the hyperparameter search, due to the imbalanced binary classes.

We split the full CGM data into a training set (75%) and a hold-out test set (25%). As above, the split was grouped at the patient level. The XGBoost model is trained on the training set (N=9,998,094) and its performance is evaluated on the test set (N=2,804,594). In the main manuscript, we focus on hypoglycaemia prediction, however, a model predicting hyperglycaemia (i.e., for this paper >270mg/dL) was also developed. Our results on detecting data drift in hyperglycaemia are consistent (albeit opposite) with the hypoglycaemia model. For clarity, we present results only for hypoglycaemia prediction in the main text.

S2: Additional covariate shift detection information

Derived from information theory, a common choice for measuring the difference between two probability distributions (defined P and Q) is the Kullback-Leiber (KL) divergence[28]. The KL distance is defined as:

$$KL(P||Q) = \sum_{x \in X} p(x) \log \frac{p(x)}{q(x)}.$$

By definition, KL(*P*||*Q*) is always non-negative and non-zero (unless the distributions are equal). KL divergence is a popular choice for covariate shift detection (e.g.,[30]), however, has weaknesses such as asymmetry and that it can extend to infinite values when there are 0 probability events[31]. The Jensen-Shannon (JS) divergence solves these weaknesses by considering the mutual information between the mixture distributions of *P* and *Q* [29]. The JS divergence is defined as:

$$JS(P||Q) = \sum_{x \in X} \left[p(x) \log \frac{2p(x)}{p(x)+q(x)} + q(x) \log \frac{2q(x)}{p(x)+q(x)} \right].$$

By considering the mutual information between the mixture distributions, the JS divergence has a finite range between 0 (i.e., *P* and *Q* are the exact same distributions) and 1 (completely different).

To quantify the degree of covariate shift, we calculate the JS divergence between the probability distributions of the training data and the period of interest (test set), for each feature. Despite the JS divergence generalising to higher dimensionality (i.e., full covariate space could be considered as one multi-variate distribution), this enables individual insight into which features are driving potential covariate shift. We then take the simple mean of the JS divergence across all covariates to summarise.

Since the KL and JS divergence are based on the probability distributions of the covariates, we are able to compute the divergence for both numerical and categorical variables (NB. in this paper we only consider numerical covariates, however, present a general solution to quantifying drift). However, the magnitude of drift values can vary with the number of bins of the covariate's probability distribution[32, 33]. The number of bins can depend both on the pre-defined cardinality of the covariate (i.e., for ordinal, categorical and certain discretized numerical variables) and the sample size of the original distribution. Prior work has defined suitable number of bins based off the sample size of the original distribution alone (e.g.,[34]), however, this doesn't allow correction for covariates with pre-defined cardinality when defining the probability distribution.

We present a general solution to normalize covariate shift values based off the training set's internal difference. To account for the variance in the magnitude of the JS divergence, we bootstrap (sampling with replacement) the training set 1000 times to create samples with equal size to the test set (i.e., period of interest). Our bootstrapped samples are drawn so that they have the same number of unique users represented as the test set. For each bootstrap, we calculate the JS divergence with the training set (excluding the drawn samples) for each covariate. Again, the mean of the JS divergence of all features is taken to give our metric. We create a distribution of JS divergence values for the 1000 bootstrap samples, which is used to normalize the test's individual JS divergence value. This is quantified by the number of standard deviations (σ) between the test set's JS divergence value and the mean of the bootstrapped distribution. By definition the bootstrap samples are consistent with the training set (i.e., $JS(P||Q) \rightarrow 0 \; as \; n_{samples} \rightarrow \infty \; and \; width_{bin} \rightarrow 0$) therefore the distance characterises the covariate specific noise. Higher positive σ values indicate greater difference and hence covariate shift, which can be directly compared between covariates and different test sets. We call this value the bootstrap normalised JS divergence.

S3: Relationship between feature distributions and SHAP explanations

In Supplementary Materials Figure 1 we plot the relationship between features values and their corresponding SHAP feature importance. By definition, SHAP provides local additive contributions to prediction where negative SHAP values correspond to the feature value lowering the probability of a

positive prediction (i.e., hypoglycemia). In the main text we only consider the magnitude of aggregated SHAP values to consider overall predictive importance. For each panel (shown for each of top 8 features), we plot the feature values against the SHAP importance. The distribution of feature values is shown in a histogram below each panel.

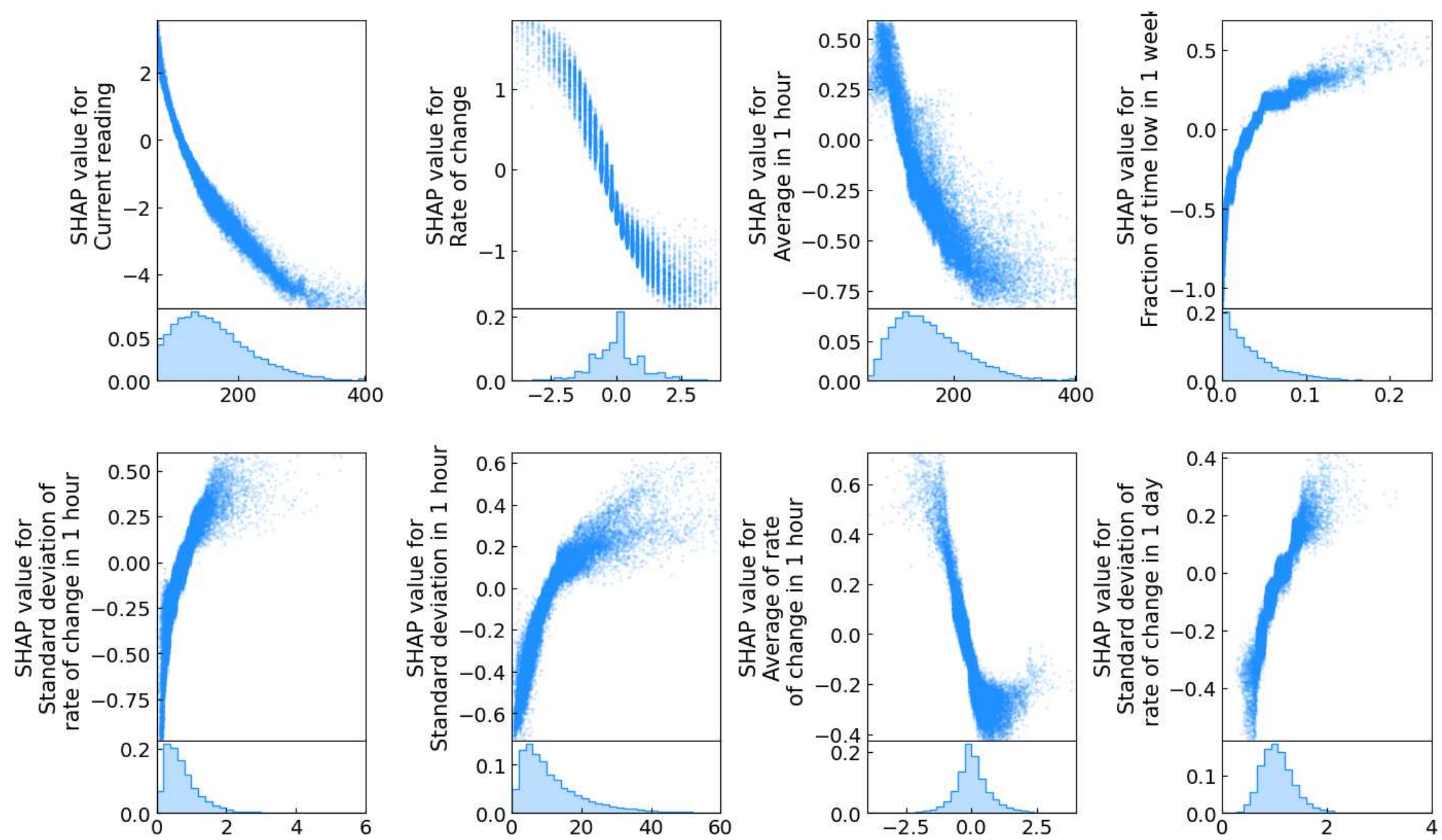

Supplementary Figure 1: Distributions of feature values vs SHAP importance values for top 8 most important features used in modelling. Under each panel shows a histogram of the feature values' distribution.

The two instantaneous features "current reading" and "rate of change" show strong negative correlations with SHAP. Practically this corresponds to the risk of hypoglycemia increasing as current readings are lower and the rate of change is more negative. This pattern is also reflected in features averaged across time periods, including "average (current reading)" and "average of rate of change". Features summarizing variability such as "standard deviation 1hour" and "standard deviation of rate of change 1hour" show positive correlations with SHAP, signifying that more variability in control increases hypoglycemia risk. Finally, the feature "fraction of time low 1week" quantifies longer-term BG trends and its positive correlation reflects that users which are more consistently low are more likely to be at risk.

S4: Variations during sensors' lifetime

S4.1 Data

In the main text we consider the isolated impact of systematic (3.4) and stochastic (3.5) noise as a simulation of sensor degradation. Here, we perform an explicit test of how the age of a sensor could impact the safety of prediction. Within the lifetime of a CGM sensor's usage, its accuracy and precision can fluctuate. When a sensor is first attached, the accuracy of the first several hours (up to 12 hours) can be impacted by irritation and inflammatory responses around the sensor area. In addition, as a sensor nears the end of its lifetime, the materials that is used to react with glucose in the blood is gradually

consumed. The CGM system counteracts this by scaling the measured glucose reaction up by a larger factor to maintain a normal level. However, the increased scaling can introduce increased random noise in the measurement. Hence, in this section, we split all individual sensor durations of individual patients into early, middle, and late periods of their lifetimes, and compare the prediction performance and explainability from datasets of only early, middle, and late sensor lifetimes.

Participants of the REPLACE dataset were instructed to replace sensors at least every 7 days, however replacements were not explicitly timestamped. We identify new sensors by requiring a >60 minutes pause in CGM readings accompanied by a finger-prick calibration test within 10 minutes before resuming readings. From the test set, all sensor durations that are longer than 3 days are then divided into three equal portions that correspond to the early, middle, and late periods in the sensor's lifetime.

S4.2 Covariate shift

In Supplementary Table 2 we show the JS divergence and normalised JS divergence for test data group by sensor age. Interestingly, all three sensor durations are more different than all of their corresponding bootstrapped subsets of the training set. This indicates there is significant difference in the distribution of the feature values between the early, middle and the late durations of sensor usage, resulting in covariate drift.

	**Early**	**Middle**	**Late**
Mean JS divergence	0.0235	0.0237	0.0238
Mean bootstrap normalised JS divergence (σ)	27.09	30.40	28.09

Supplementary Table 2: Mean JS distance and normalised JS divergence (i.e., number of sigma relative to bootstrapping) for test sets grouped by sensor age.

S3.3 Predictive Performance

In Supplementary Figure 2 (top) we show how model performance changes with sensor age. Comparing this figure to other scenarios in the main text; the variation in performance due to sensor age is negligible. This indicates that in general the model is robust across sensor lifetimes against the covariate drift detected from our JS distance metrics. Amongst the small amount of performance variation, the earliest third of sensor lifetime has an elevated fraction of positive class (i.e., hypoglycemia).

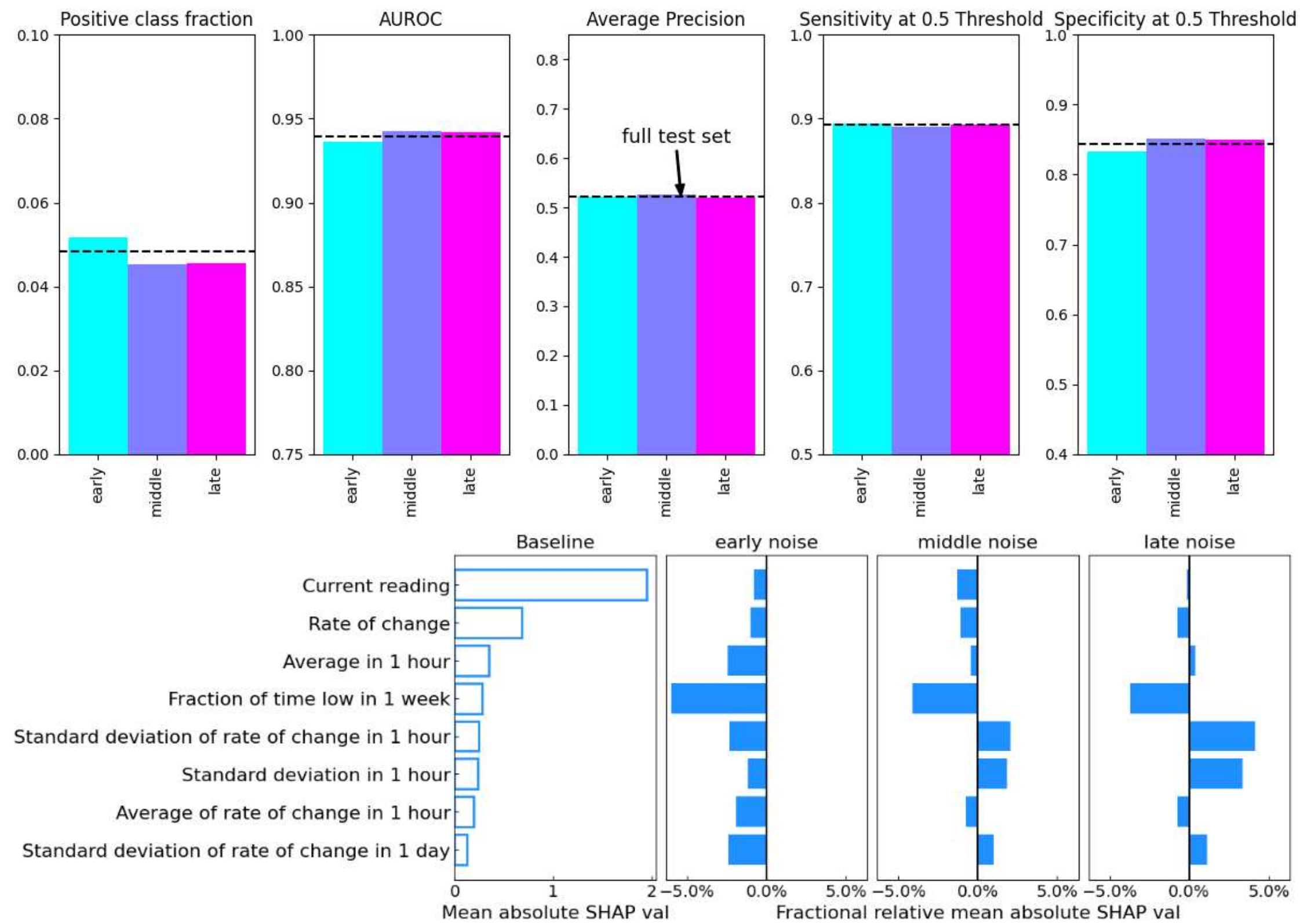

Supplementary Figure 2: (Top) Summary of model performance for increasing sensor age; (left-to-right) positive class fraction, AUROC, Average Precision, Sensitivity and Specificity (dichotomised at 0.5 probability). The dashed black line shows the baseline value for the unadjusted test set. (Bottom) Feature importance for prediction as measured by SHAP. The left-most panel shows the baseline importance with all other panels showing the relative change.

S4.4 Explanations

In Supplementary Figure 2 (bottom) we show the variation in feature importance with sensor age. We find no discernable trends, other than variation features (e.g., standard deviation over past hour) increasing in importance for older sensors. This could suggest a slight increase in sensor noise, in-keeping with our results in 3.5.

S5: Effect of long-term data drift

In this section we consider the practical implementation of drift detection for individuals using CGM. While our scenarios demonstrate isolated tests of drift, in practice data will likely be a combination of multiple effects (i.e., both sensor and user). Here, we consider how the performance of our model drifts in the long-term up-to 150 days after the original training of the model. This test reflects the real-use situations when an increasing span of time has elapsed since the last training of the model. By tracking changes in the input CGM data, model prediction performances and relative feature importance, the impact of data drift can be quantified and understood. Therefore, the results can inform on the frequency or thresholds

based on changes in the input CGM data and/or prediction performance at which the model has to be retrained.

S5.1 Data

We first reserve the first 150 days' worth of cleaned (usage fraction > 0.8) CGM data from all individuals in REPLACE as the training and hold-out (baseline) test sets, while the remaining data are split into individual 30-days sets. We follow the same procedure as Section 2.2 for train-test split, hyperparameter tuning and model training. We bin our remaining test data in one-month (30 day) subsets up-to 150 days after our model training data ends.

S5.2 Covariate shift

S5.3 Predictive performance

Supplementary Figure 3 shows the receiver operating characteristic (left) and precision-recall (right) curves of our hypoglycemia model, evaluated on a random holdout (grey) and temporal holdouts (coloured) in 30-day increments after the training period. Overall, we find a very small effect that model performance degrades over time (120-150 days after training has the lowest metric values). Following the performance of individual users over time shows more pronounced effects.

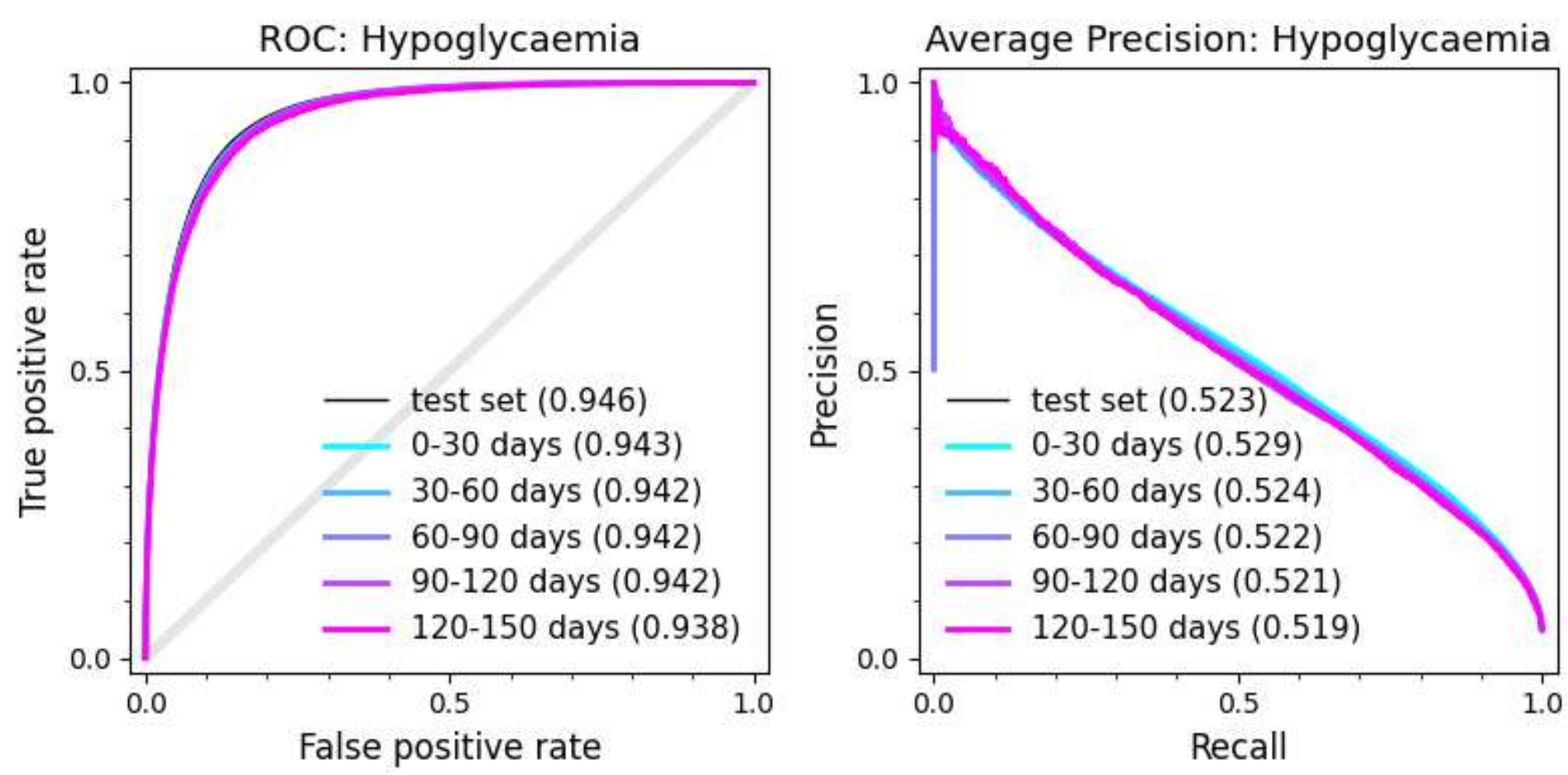

Supplementary Figure 3: ROC (left) and precision-recall (right) for our hypoglycemia model trained on only the first 150 days of CGM use for each user. Performance is shown for a hold-out test set (grey) using a random holdout within the first 150 days of data in-comparison to temporal hold-out test sets in 30-day increments after the 150-day training period.

This is further confirmed in Supplementary Figure 4 (top) which shows a small degradation in performance in AUROC and Average Precision over time. This is more notably seen by a lower sensitivity, while specificity shows no particular trend.

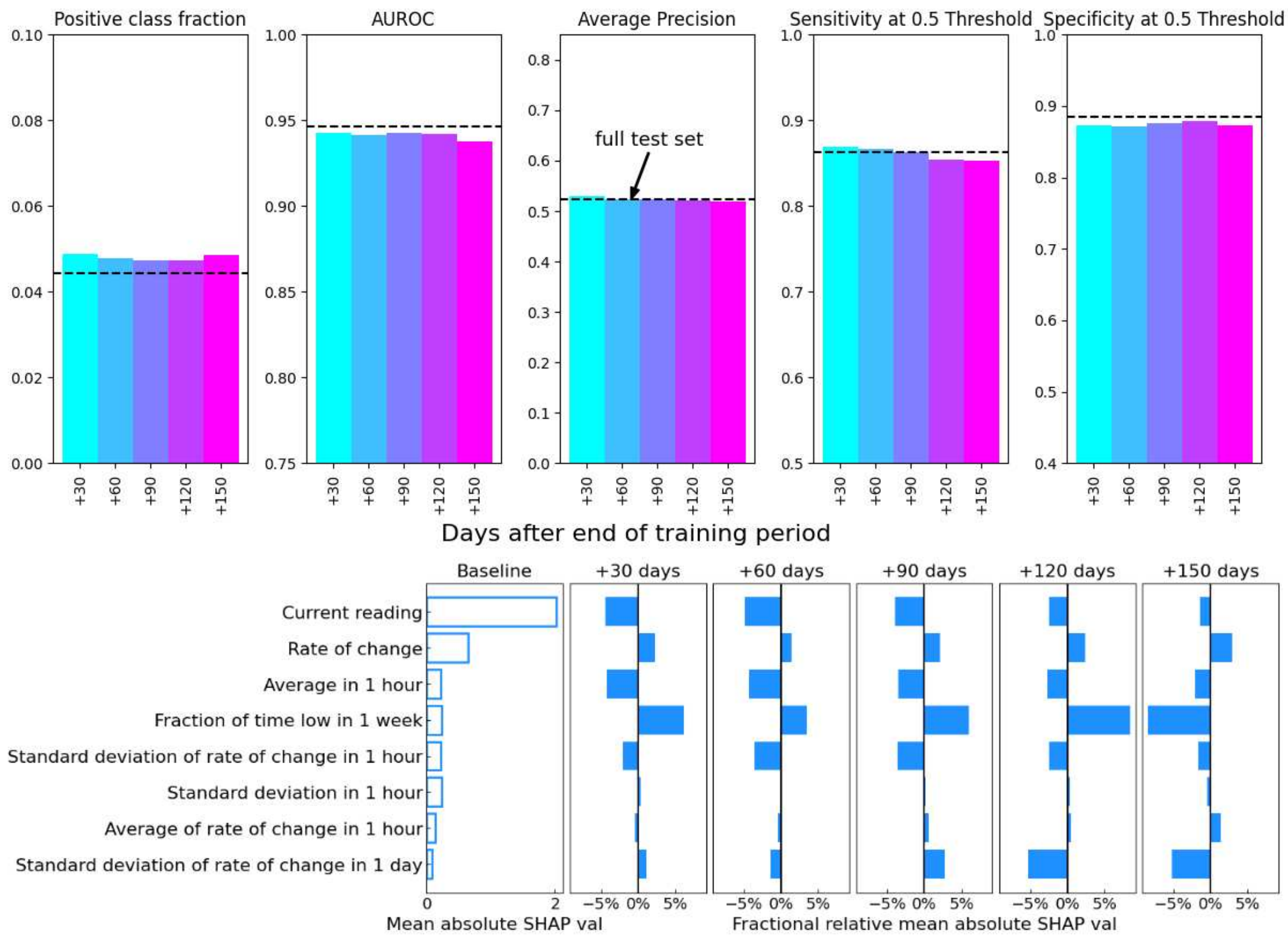

Supplementary Figure 4: Summary of model performance for tests of increasing distance from training time (and baseline); (left-to-right) positive class fraction, AUROC, Average Precision, Sensitivity and Specificity (dichotomised at 0.5 probability). (Bottom) Feature importance for prediction as measured by SHAP. The left-most panel shows the baseline importance with all other panels showing the relative change.

S5.4 Explanation

Supplementary Figure 4 (bottom) shows how feature importance changes over time with increasing time after model training. Instantaneous and short-term features (e.g., current reading, rate of change, average in 1 hour, standard deviation of rate of change in 1 hour) show consistent importance, with variance being driven by longer term features (e.g., fraction of time low in 1 week, standard deviation of rate of change in 1 day). This suggests that the engineering of longer-term features should be consider if the model is retrained.
